# Optimization of Aminoimidazole Derivatives as Src Family Kinase Inhibitors

**DOI:** 10.3390/molecules23092369

**Published:** 2018-09-17

**Authors:** Cinzia Maria Francini, Francesca Musumeci, Anna Lucia Fallacara, Lorenzo Botta, Alessio Molinari, Roberto Artusi, Laura Mennuni, Adriano Angelucci, Silvia Schenone

**Affiliations:** 1 Rottapharm Biotech S.r.l. Valosa di Sopra N9Street, 20900 Monza, Italy; cinziamariafrancini@libero.it (C.M.F.); roberto.artusi@rottapharmbiotech.com (R.A.); laura.mennuni@rottapharmbiotech.com (L.M.); 2 Department of Pharmacy, University of Genova, Viale Benedetto XV 3, 16132 Genova, Italy; francesca.musumeci@unige.it; 3 Department of Biotechnology, Chemistry and Pharmacy, Via Aldo Moro 2, 53100 Siena, Italy; al.fallacara@gmail.com (A.L.F.); molinari.a2@gmail.com (A.M.); 4 Department of Biological and Ecological Science, University of Tuscia, 01100 Viterbo, Italy; lorenzo.botta@unitus.it; 5 Department of Applied Clinical Science and Biotechnology, University of L’Aquila, Via Vetoio Coppito, 67100 L’Aquila, Italy; adriano.angelucci@univaq.it

**Keywords:** aminoimidazole, SFK inhibitors, neuroblastoma, anticancer, tyrosine kinases

## Abstract

Protein kinases have emerged as crucial targets for cancer therapy over the last decades. Since 2001, 40 and 39 kinase inhibitors have been approved by FDA and EMA, respectively, and the majority are antineoplastic drugs. Morevoer, many candidates are currently in clinical trials. We previously reported a small library of 4-aminoimidazole and 2-aminothiazole derivatives active as Src family kinase (SFK) inhibitors. Starting from these results, we decided to perform an optimization study applying a mix and match strategy to identify a more potent generation of 4-aminoimidazoles. Firstly, a computational study has been performed, then compounds showing the best predicted docking scores were synthesized and screened in a cell-free assay for their SFK inhibitory activity. All the new chemical entities showed IC_50_s in the nanomolar range, with 2–130 fold increased activities compared to the previously reported inhibitors. Finally, the most active compounds have been tested on three cancer cell lines characterized by Src hyperactivation. Compounds **4k** and **4l** showed an interesting antiproliferative activity on SH-SY5Y neuroblastoma (NB) cell line. In this assay, the compounds resulted more potent than dasatinib, a tyrosine kinase inhibitor approved for the treatment of leukemias and in clinical trials for NB.

## 1. Introduction

Protein kinases are a large class of enzymes (to date 518 members have been identified) which are involved in different phases of the cell life [1]. These proteins are overexpressed and/or hyperactivated in several diseases, including cancer, neurodegenerative disorders and inflammation [2]. For this reason, protein kinases have become a thoroughly studied target in medicinal chemistry and, to date, 40 and 39 kinase inhibitors have been approved by the FDA and EMA, respectively, and the majority are antineoplastic drugs [3].

Protein kinases phosphorylate tyrosine, serine, and threonine residues in protein substrates and on this basis are classified as tyrosine kinases (TKs) and serine/threonine kinases (STKs) [4]. Human TKs are further divided in two main families: receptor tyrosine kinases and non-receptor tyrosine kinases. Into the latter, Src family kinases (SFKs) are the biggest subfamily of enzymes. This class includes nine highly homologous members, i.e., Src, Fyn, Yes, Blk, Yrk, Fgr, Hck, Lck, and Lyn. All SFKs present a common structure characterized by a N-terminal Src homology domain (SH4), a ‘unique’ region—different among SFK members, two Src homology domains (SH2 and SH3), a catalytic domain (SH1), and a short C-terminal tail. The catalytic domain constitutes the core of the enzyme since it possesses the kinase activity. SH1 presents a bilobal structure, with a small *N*-terminal lobe and a large C-terminal lobe, linked by a flexible chain, named hinge region [5]. At the interlobe cleft there are the ATP- and substrate-binding sites. Under basal conditions, SFKs present a close and inactive conformation that prevents ATP and substrate binding. After phosphorylation of a specific tyrosine residue (Tyr419 in human Src) in the C-terminal lobe by upstream kinases, a structural rearrangement occurs and results in a flip to an open and active conformation. At this point, the enzyme is able to bind ATP and the opportune peptide substrate, that will be phosphorylated [6,7]. SFKs are involved in the regulation of different signal transduction pathways such as growth, proliferation, differentiation, migration, metabolism, and apoptosis and, as reported for kinases in general, their overexpression and/or hyperactivation have been shown in many types of tumors [8,9]. Interestingly, despite the high homology among SFK members, different pathological implications have been detected inside the family. For instance, Fyn plays a key role in brain pathologies, such as Alzheimer’s disease [10], whereas Hck, Fgr, and Lyn are the main SFK members involved in inflammation [11].

**Figure 1:**
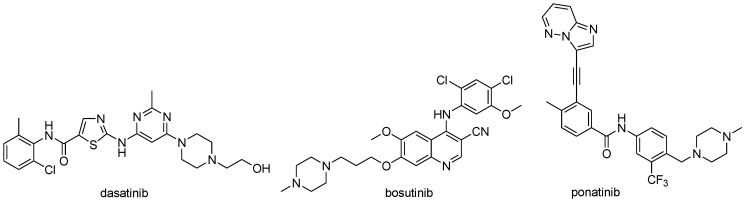
Structures of approved SFK inhibitors dasatinib, bosutinib, and ponatinib.

To date, three molecules active as SFK inhibitors have been approved for clinical use, i.e., dasatinib, bosutinib, and ponatinib (Figure 1) [3,12,13]. Anyway, these compounds are not selective SFK inhibitors, but also inhibit other kinases. Dasatinib (Sprycel^®^, Bristol-Myers Squibb, approved in 2006), and bosutinib (Bosulif^®^, Wyeth, approved in 2012), are two dual Src/Bcr-Abl (another non-receptor TK) inhibitors which bind the enzymes in their active conformation. Dasatinib potently inhibits all nine members of SFKs [14] and, in detail, a KINOMEscan analysis published by Davis et al. in 2011 [15] showed that it possesses K_d_ values of 0.21, 0.30, 0.53, and 0.79 nM on c-Src, Yes, Lyn, and Fyn, respectively. The compound also inhibits other TKs, including Kit, PDGFR, ephrin A receptor kinase, and the Tec kinase Btk [16]. Dasatinib has been approved for the treatment of chronic myeloid leukemia (CML) and acute lymphoblastic leukemia Philadelphia chromosome-positive (ALL Ph+), and is currently in clinical trials for NB and other solid tumors [17]. NB is an extracranial solid tumor of childhood often characterized by poor prognosis and for which an effective treatment still lacks. Bosutinib has been approved for the treatment of CML Ph+ with resistance or intolerance to prior therapy, and in December 2017, the FDA granted accelerated approval as first line therapy [18]. Ponatinib (Iclusig^®^, Ariad Pharmaceuticals, approved in 2012) is a so-called “pan-kinase” inhibitor, since it is active against many kinases, including SFKs, Bcr-Abl, VEGFR, PDGFR, and Ret. Ponatinib binds Src and Bcr-Abl in their inactive conformation. It has been approved for CML or ALL Ph+, in particular for resistant T315I-positive CML or T315I-positive, Ph+ ALL [19]. 

Although the majority of protein kinase inhibitors occupy the ATP binding site, few compounds are able to bind pockets that are far from the catalytic cleft and act as allosteric inhibitors [20]. The complexity in regulating kinases’ activity offers many potential routes for pursuing their inhibition and, despite the initial concerns about the possibility to identify potent and selective kinases inhibitors, significant advances have been made over the past two decades [21,22,23]. 

As widely reported in the literature, kinase inhibitors usually have heteroaromatic scaffolds capable of interacting with the hinge region [8]. Therefore, the synthesis of new hinge interacting moieties is crucial to obtain new molecules that may be selective for one or a few kinases and endowed with a high inhibitory potency. 

Zhang et al. described in detail the binding site of different kinase inhibitors and showed many heterocyclic ring systems that occupy the purine binding site [24]. The strength of the binding between these heterocyclic moieties and the target kinase is due to electrostatic interactions; in particular, some hinge binders were designed to establish one to three hydrogen bonds to the hinge region.

**Figure 2:**
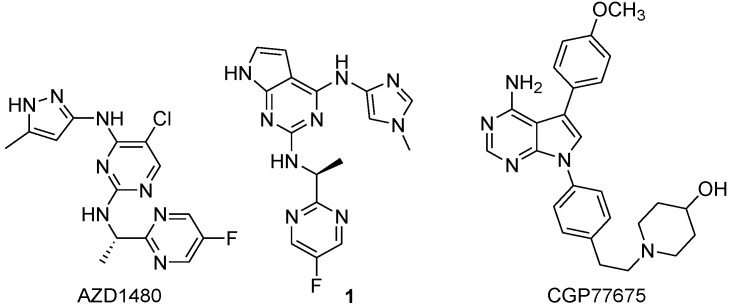
Molecular structures of AZD1480, **1**, and CGP77675.

From an extensive literature study, the 4-aminoimidazole and the 2-aminothiazole rings emerged as interesting starting entities for the development of new ATP pocket binders. The 4-aminoimidazole moiety has been first explored by AstraZeneca [25], who highlighted the ability of this scaffold to bind the Janus kinase hinge region. The authors compared their clinical candidate AZD1480 (a Jak2 inhibitor with an IC_50_ of 58 nM, Figure 2), containing the 3-aminopyrazole moiety, with the related 4-aminoimidazole derivative (IC_50_ value of 120 nM towards Jak2), and found that this bioisosteric substitution could be an effective replacement for the 3-aminopyrazole ring. The subsequent modulation of this first hit led to the discovery of the potent and orally bioavailable Jak2 inhibitor **1** (IC_50_ < 3 nM, Figure 2) [25].

On the other hand, the 2-aminothiazole represents the hinge binder moiety of dasatinib, the potent SFK inhibitor already reported. 

**Figure 3:**
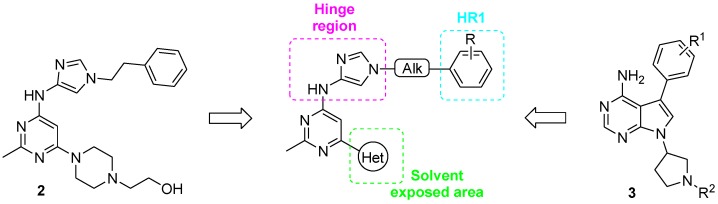
The mix and match strategy applied for the design of NCEs.

In a previous work, we described the synthesis and the biological evaluation of a set of 4-aminoimidazole and 2-aminothiazole derivatives as SFK inhibitors [26]. The 4-aminoimidazole ring was demonstrated to be an effective hinge binder for c-Src kinase, suggesting that this moiety, properly functionalized, is a good replacement for the aminothiazole ring [26]. Indeed, the most active compound **2** (Figure 3) showed IC_50_ values of 220, 689, 1300, and 167 nM for the isolated enzymes Src, Fyn, Lyn, and Yes, respectively (Table 1), and resulted active on SH-SY5Y neuroblastoma (NB) cell line with an IC_50_ of 25 μM. On the other hand, **2** showed a weak activity on K562 CML cell line, possessing an IC_50_ > 25 μM. As a continuation of our work, herein we present an optimization study aimed at obtaining a new generation of (1*H*-imidazol-4-yl)-pyrimidin-4-yl-amines endowed with a higher affinity towards SFKs and a stronger activity on cells compared to compounds previously reported by us. 

## 2. Results and Discussion

### 2.1. Docking Studies

Starting from our hit **2** [26] and **3** (a potent Src inhibitor) [27], a mix and match strategy combined with the use of computational tools has been applied for the identification of new chemical entities (NCEs) acting as ATP pocket binders (Figure 3). As a first step towards a better understanding of the molecular determinants for the inhibitory activity of this class of compounds against Src, a molecular docking simulation has been performed on NCEs and the upcoming results were compared with the best aminoimidazole hit **2** reported in our previous work [26]. In particular, more than one hundred NCEs were designed and docked into the ATP binding site of Src by using the 3G5D X-ray structure [28]. The mix and match strategy has been applied in order to (i) introduce different alkyl groups as linkers to investigate the steric hindrance allowed around the hinge region; (ii) introduce hydrophobic and hydrophilic moieties interacting with the hydrophobic region I (HRI) to improve the primary activity by achieving electrostatic interactions that would be missing with an unsubstituted phenyl group; (iii) replace the solvent exposed substituent with different types of heterocycles. 

Docking studies were performed by means of Glide [29] software and the reliability of the applied protocol was first assessed by reproducing the experimental binding mode of two known inhibitors of Src: dasatinib (PDB code: 3G5D) [28] and CGP77675 (PDB code: 1YOL [30], Figure 2). Compounds were drawn, minimized and finally docked into the ATP-binding site of Src (3G5D) [28]. 

**Figure 4:**
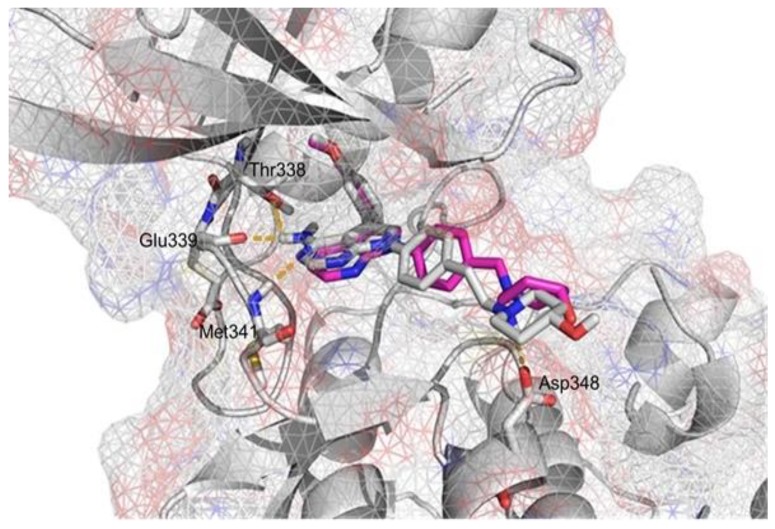
Docking pose of CGP77675 (gray sticks) in the ATP-binding site of c-Src. The X-ray structure has been overlapped for reference (magenta sticks). For sake of clarity, only a few residues are displayed in gray sticks. Hydrogen bonds are shown as yellow dashes.

As a result, the program was able to reproduce the experimental poses of the two compounds with a RMSD of 0.55 Å suggesting that the docking procedure could be reliable to predict the binding mode of our NCEs (Figure 4).

**Figure 5:**
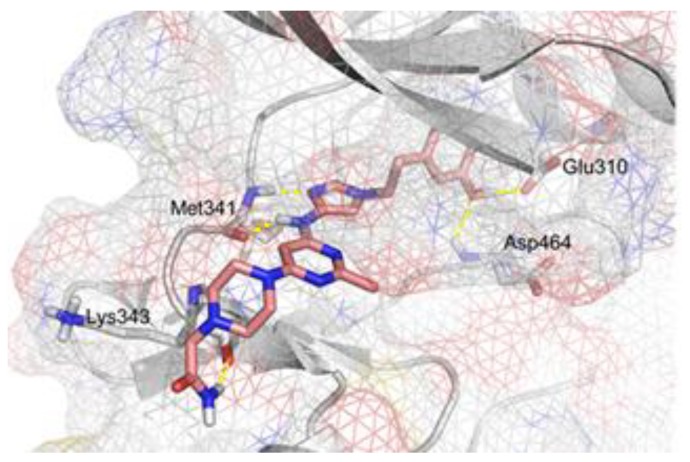
Docking pose of **4j** (pink sticks) in the ATP-binding site of c-Src. Hydrogen bonds are shown as yellow dashes.

Compounds **4a**–**g** (Table 1), showing the best predicted docking scores, are characterized by a series of polar moieties in the solvent exposed region, and hydroxyl or methoxyl groups on the phenyl ring of the *N*-1-(2-phenylethyl)-1*H*-imidazol-4-yl side chain. These substitution patterns have been selected with the aim of improving the water solubility and getting further insights into this class of inhibitors. All compounds showed a similar interaction pattern characterized by two hydrogen bonds involving the imidazole nucleus and the hinge region: one between the N3 and the NH backbone of Met341, and one between the 4-NH and the CO backbone of Met341. The phenyl ring was located into the HR1, forming Van der Waals interactions with hydrophobic amino acids of this region. Moreover, the new *N*-[1-(2-phenylethyl)-1*H*-imidazol-4-yl]pyrimidinamines interacted with two different residues in HR1 and in solvent exposed area. The best compounds in terms of docking scores show a *meta* or *ortho* hydroxyl group on the phenyl ring and an amide, ester, or carbamate group in N4 position of the piperazine chain. In detail, the pose example of compound **4j** (GB = −11.33 kcal/mol) has been reported in Figure 5: the *meta* hydroxyl group acts as both H-bond donor and acceptor in the interactions with Glu310 and Asp464 respectively. The *ortho* substituted derivative **4g** (GB = −11.23 kcal/mol) establishes a hydrogen bond interaction with Asp464 belonging to the DFG motif. 

As shown in Table 1, compounds **4g** and **4j**, having the highest values of docking score, are predicted to be the most active compounds on the selected kinase, while compounds **4b** and **4d** (−7.266 and −8.336 kcal/mol respectively) resulted as the least active ones. 

### 2.2. Chemistry

**Scheme 1:**
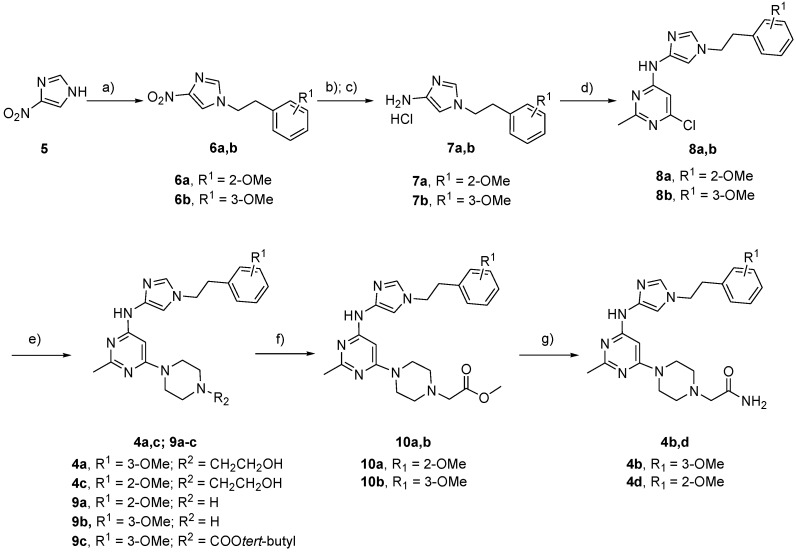
Synthesis of final compounds **4a**–**d** and intermediates **9a**–**c**, **10b**. Reagents and conditions: (**a**) BrCH_2_CH_2_C_6_H_4_R^1^, K_2_CO_3_, CH_3_CN, 60 °C, overnight; (**b**) H_2_, Pd/C, EtOH, rt, 2 h, atmospheric pressure; (**c**) HCl in EtOH 2M, rt, 30 min; (**d**) 4,6-dichloro-2-methylpyrimidine, DIPEA, DMSO, 50 °C, overnight; (**e**) Boc-piperazine or piperazine or 2-(piperazin-1-yl)ethanol, DIPEA, DMSO, 110 °C, 4 h under microwave irradiation; (**f**) methyl bromoacetate, TEA, DMF, rt, 10 min; (**g**) ammonia 7N in MeOH, 100 °C, 3 h, under microwave irradiation.

The best predicted derivatives, in respect to our previous reported hit **2**, were selected to be synthesized and tested (Table 1). **4b** and **4d** were also prepared as negative controls in enzymatic assays. Compounds **4a**–**d**, bearing a hydrophobic group –OMe exposed to HR1, were first synthesized (Scheme 1) [26]. The commercially available 4-nitro-1*H*-imidazole **5** was regioselectively functionalized using opportune alkylating agents to give intermediates **6a**,**b** in high purity and yield [26,31]. Subsequent palladium mediated hydrogenation of **6a**,**b** afforded the corresponding amino derivatives as free bases, which were immediately converted to hydrochloride salts, since the compounds are unstable as free bases, to yield derivatives **7a**,**b**. Compounds **7a**,**b** were regioselectively coupled with the commercially available 4,6-dichloro-2-methylpyrimidine at 50 °C in the presence of *N*,*N*-diisopropylethylamine (DIPEA) to afford intermediates **8a**,**b**.

**Scheme 2:**
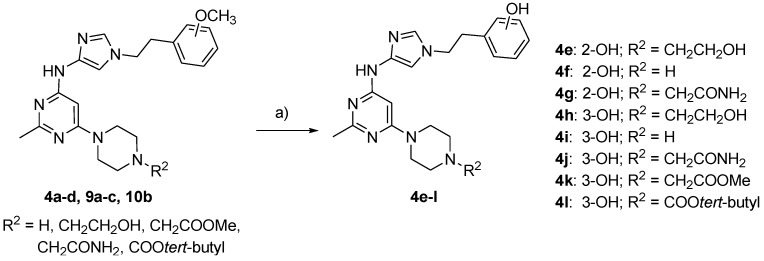
Synthesis of final compounds **4e**–**l**. Reagents and conditions: (**a**) Sodium methanethiolate, DMF, 140 °C, 2–6 h under microwave irradiation.

The intermediates **8a**,**b** were reacted with the appropriate piperazines at 110 °C under microwave irradiation in the presence of DIPEA to afford the final compounds **4a**,**c** and the intermediates **9a**–**c**, used for the synthesis of phenol derivatives **4f**,**i**,**l** described in the Scheme 2. Furthermore, **9a**,**b** were reacted with methyl bromoacetate to obtain intermediates **10a**,**b**, that were treated with ammonia 7N in MeOH to yield the final compounds **4b**,**d**, according to the procedure used by Novartis (Scheme 1) [27].

Finally, methoxy derivatives **4a**–**d**, **9a**–**c**, and **10b** were treated with sodium methanethiolate in DMF to obtain the corresponding phenol derivatives **4e**–**l** (Scheme 2).

### 2.3. Enzymatic Assays

Compounds **4a**–**l** have been tested against the isolated Src enzyme and showed IC_50_ values in the nanomolar range. Derivatives **4e**–**l** were found more active on Src than the previous reported hit **2**, showing IC_50_ values from 93 nM to 40 nM. In particular, compounds **4g**, **4j** and **4k**, bearing a hydroxyl group in the *ortho* or *meta* positions of the phenyl ring and an amide or methylester substituent as side chain, resulted to have the highest inhibitory activity (IC_50_ values of 40 nM). On the other hand, the methoxy derivatives **4a**–**d** are less potent on Src (IC_50_ values 225–1533 nM) compared with the phenolic derivatives, confirming the importance of the hydroxyl group, as predicted by modeling studies. In addition, all new compounds were tested for their activity against other members of SFKs. As expected, the most promising compounds were also potent inhibitors of Yes, Lyn, and Fyn with IC_50_ values in the range 3–73 nM. These results confirmed the hypothesis that the 4-aminoimidazole template, properly decorated, is an effective hinge binder for SFKs and has a good/high in vitro potency on these enzymes. 

### 2.4. Cellular Assays

**Figure 6:**
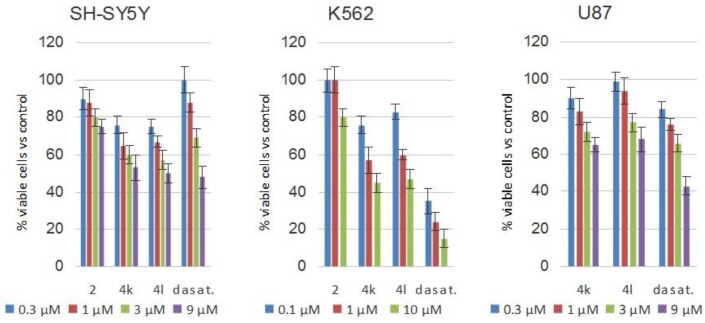
Antiproliferative effect of **4k** and **4l** evaluated by in vitro analysis of percentage of viable cells after 72 h of incubation with SH-SY5Y, K562, and U87 cells. Dasatinib was used as reference compound. Antiproliferative activity of compound **2** in SH-SY5Y and K562 cells has been also reported. Percentage was calculated respect to the number of cells treated with the vehicle (100%, bar not shown in the graph).

Starting from these promising results in enzymatic assays, we decided to test NCEs **4** on K562 CML and SH-SY5Y NB cell lines, to evaluate if they are endowed with an increased antiproliferative activity compared with the hit compound **2**. A hyperactivation of SFKs has been detected in both K562 and SH-SY5Y cell lines [9,32,33]. Cells were treated with increasing concentrations of compounds and cell proliferation was measured by counting viable cells after 72 h of incubation. Dasatinib and **2** were used as reference compounds. In Figure 6 we show the activity of **4k** and **4l** that demonstrated, in comparison with the other NCEs (see Supplementary Materials, Figure S1), the best antiproliferative activity on SH-SY5Y cells. In detail, **4k** and **4l** possess IC_50_ values of 8.6 and 7.8 μM, respectively, and show a more than 2-fold increased activity compared to the hit compound **2**. Importantly, in NB cells, **4k** and **4l** exerted an antiproliferative effect similar or higher than dasatinib. The activity of these compounds could be due not only to Src inhibition, but also to their effect on Fyn and Lyn, both involved in NB development [34]. Furthermore, both the compounds showed a similar activity on K562 cells, with an antiproliferative effect comparable with the one observed on NB cells. In fact, compounds **4k** and **4l** show IC_50_ values of 11.7 and 18.9 μM, respectively, and are more active than **2**, but less active than dasatinib (Figure 6). 

On the basis of the exciting activity of **4k** and **4l** on NB cell lines in comparison with dasatinib, we decided to test NCEs also on U87 glioblastoma multiforme (GBM), another tumor characterized by Src hyperactivation (Figure 6) [35]. Compounds **4k** and **4l** showed IC_50_s of 12.6 and 13.3, respectively, but, unfortunately, resulted less active than dasatinib. A possible explanation could be the multidrug resistance mechanisms that GBM cells usually carry: an example is the overexpression of membrane channels (ABCB1) that are able to pump different kind of drugs out of the cells [36]. In Supplementary Materials, Figure S1, the activity of other NCEs on U87 GBM cell line are reported. 

In conclusion, a small library of aminoimidazole derivatives was synthesized and screened in a cell-free assay for their SFK inhibitory activity. Enzymatic assays showed an increase in potency against isolated Src (from a micromolar range of our previously reported compounds [13] to nanomolar of the new molecules), with an exceptional increase in potency also against other SFK members. Furthermore, the most active inhibitors have been tested on three different cancer cell lines, i.e., NB, GBM, and CML cell lines. Interestingly, compounds **4k** and **4l** showed good antiproliferative activity in the SH-SY5Y NB cell line. In this assay the compounds resulted more potent than dasatinib, a TKI inhibitor which is currently in clinical trials for NB [17].

Further studies on this class of compounds will be focused on the improvement of the ADME properties, with the aim of obtaining more potent compounds in cell assays.

## 3. Materials and Methods 

### 3.1. Computational Studies

#### 3.1.1. Protein Preparation

Crystal structures of c-Src in complex with dasatinib and CGP77675 (PDB IDs:3G5D [28] and 1YOL [30]), were retrieved from the RCSB Protein Data Bank. After removal of bound ligands, the proteins were prepared by using the Protein Preparation Wizard [37] workflow (Schrodinger Suite). In particular, all water molecules were deleted, hydrogen atoms were added, and partial charges assigned. In addition, the ionization and tautomeric states of His, Asp, Glu, Arg, and Lys were adjusted to match pH 7.4. Next, optimization of the hydrogen bonding network was obtained by reorienting hydroxyl and thiol groups, amide groups of Asn and Gln, and the His imidazole ring. Finally, the systems were refined by running a restrained minimization (OPLS3 force field) which was stopped when the RMSD of heavy atoms reached 0.30 Å, the default limit.

#### 3.1.2. Ligands Preparation

Dasatinib, CGP77675 and all our NCEs were drawn and minimized using Maestro 11 (Schrödinger, LLC, New York, NY, USA) and MacroModel (Schrödinger, LLC, New York, NY, USA) (Schrodinger Suite), respectively. Furthermore, LigPrep (Schrödinger, LLC, New York, NY, USA) was used to predict ionization and tautomeric states for the ligands using pH 7 ± 0.4.

#### 3.1.3. Molecular Docking

Docking simulations were performed using the Glide program (Schrödinger, LLC, New York, NY, USA) [38,39] within the ATP binding site of Src (3G5D). A grid box of default size was centered on the X-ray ligand. No constraints were included during grid generation while rotation of the hydroxyl groups was allowed only for those residues closer to the ligand. After grid preparation, NCEs were flexibly docked and scored using the Glide standard-precision (SP) mode, treating the proteins as rigid. Docking experiments were performed using 0.80 factor to scale vdW radii of the nonpolar ligand atoms with a partial atomic charge of <0.15.

### 3.2. Chemistry

#### 3.2.1. General Information

Reagents used in the following examples were commercially available from various suppliers (for example Sigma-Aldrich (Milan, Italy), Fluorochem (Hadfield, UK) or Apollo Scientific (Bredbury Stockport, UK)) and used without further purification. Anhydrous reactions were run under a positive pressure of dry N_2_. 

^1^H NMR spectra and ^13^C NMR were recorded on Bruker 400 MHz NMR spectrometer (Milan, Italy) using deuterated DMSO (DMSO-*d*_6_). Chemical shifts are reported in ppm (δ) using the residual solvent as the internal standard. Splitting patterns describe apparent multiplicities and are designated as s (singlet), d (doublet), dd (doublet of doublets), t (triplet), m (multiplet), and br s (broad singlet). 

IR spectra were recorded on Perkin Elmer Spectrum 100 FT-IR spectrometer (Milan, Italy).

The accurate masses were measured by the LTQ-Orbitrap XL (Thermo Scientific, Bremen, Germany) mass spectrometer interfaced with an electrospray ionization (ESI) (Thermo Fisher Scientific, Waltham, MA, USA) source characterized by spray voltage 4.5 kV, nitrogen as sheath gas (10 a.u). The resolution of accurate masses is 30,000. MS/MS spectra were recorded with an isolation windows of 2 mass units, collision energy of 15, 16 or 17 V and Helium as collision gas. UPLC spectra were performed on a Waters Acquity UPLC-SQD (Waters, Milford, MA, USA) instrument using an Acquity UPLC-BEH C18 column (1.7 µM, 50 × 2.1 mm) eluting with a gradient mixture of H_2_O + 0.1% formic acid-acetonitrile + 0.1% formic acid or ammonium bicarbonate 10 mM (pH = 9) and acetonitrile.

Flash silica gel chromatography was performed on Biotage automatic flash chromatography systems (Uppsala, Sweden) (Isolera or SP1) using Biotage SNAP HP silica cartridges or Biotage SNAP KP-NH cartridges. Reverse phase chromatography was performed on a Biotage automatic flash chromatography system (Isolera) using Redisep Gold C-18Aq cartridges. Purification was also done by SPE with SCX cartridges. Reactions were monitored by thin-layer chromatography on 0.25 mm E. Merck silica gel plates (60F-254), visualized with UV light. Melting points (Mp) were determined with a Büchi B-540 apparatus.

Microwave irradiation experiments were conducted on a Biotage Initiator microwave reactor. 

#### 3.2.2. General Procedure for the Synthesis of 1-(2-Phenethyl)-4-nitro-1*H*-imidazole Derivatives **6a**,**b**

The opportune alkyl bromide (916 mg, 4.26 mmol) was added to a solution of 4-nitro-1*H*-imidazole **5** (438 mg, 3.87 mmol) in acetonitrile (3 mL) and potassium carbonate (803 mg, 5.81 mmol). The resulting mixture was heated at 60 °C overnight. The reaction mixture was then filtered, and the filtrate was concentrated in vacuum, leaving a yellow solid. The desired product was recovered from this residue by normal phase column chromatography on a cartridge Biotage HP-SiO_2_ (50 g) column primed with DCM only. The column was then run for 4CV with DCM and then changed to DCM/MeOH 9:1 over 5CV. 

*1-[2-(2-Methoxyphenyl)ethyl]-4-nitro-1H-imidazole* (**6a**): White solid, 82% yield, mp: 100–102 °C; IR: ν = 1338 (NO) cm^−1^; ^1^H NMR (400 MHz, DMSO-*d*_6_) δ = ppm 3.11 (t, *J* = 7.34 Hz, 2H), 3.75 (s, 3H), 4.28 (t, *J* = 7.34 Hz, 2H), 6.84 (t, *J* = 7.34 Hz, 1H) 6.93–7.06 (m, 2H), 7.19–7.26 (m, 1H), 7.67 (s, 1H), 8.30 (s, 1H); ^13^C NMR (100 MHz, DMSO-*d*_6_) δ = ppm 158.8, 146.2, 138.7, 129.9, 128.4, 126.8, 121.5, 120.4, 112.9, 56.8, 49.6, 28.6; HRMS (ESI): *m*/*z* calcd. for C_12_H_13_N_3_O_3_ 248.9567 [M + H]^+^, found 248.9542. 

*1-[2-(3-Methoxyphenyl)ethyl]-4-nitro-1H-imidazole* (**6b**): Pale yellow solid, 91% yield, mp: 95–97 °C; IR: ν = 1339 (NO) cm^−1^; ^1^H NMR (400 MHz, DMSO-*d*_6_) δ = ppm 3.08 (t, *J* = 7.34 Hz, 2H), 3.72 (s, 3H), 4.33 (t, *J* = 7.34 Hz, 2H), 6.69–6.82 (m, 3H), 7.20 (m, 1H), 7.74 (s, 1H), 8.38 (s, 1H); ^13^C NMR (100 MHz, DMSO-*d*_6_) δ = ppm 159.5, 146.2, 140.7, 138.7, 129.1, 121.4, 120.4, 115.4, 112.6, 56.0, 49.5, 33.8; HRMS (ESI) *m*/*z* calcd. for C_12_H_13_N_3_O_3_ 248.9567 [M + H]^+^, found 248.9588.

#### 3.2.3. General Procedure for the Synthesis of 1-(2-Phenylethyl)-1*H*-imidazol-4-amines **7a**,**b**

1-(2-Phenethyl)-4-nitro-1*H*-imidazole derivatives **6a**,**b** (544 mg, 2.2 mmol) were dissolved in EtOH and Pd/C (10 wt %, 96 mg, 0.09 mmol) was added. The mixture was subjected to hydrogenation at atmospheric pressure for 2 h at room temperature. The mixture was filtered to remove the catalyst and then an excess of 1.25 M HCl in EtOH was added. The resulting solution was stirred for 30min and concentrated under reduced pressure to give compounds **7a**,**b**.

*1-[2-(2-Methoxyphenyl)ethyl]-1H-imidazol-4-aminehydrochloride* (**7a**): Yellow oil, quantitative yield; ^1^H NMR (400 MHz, DMSO-*d*_6_) δ = ppm 3.04 (t, *J* = 7.09 Hz, 2H), 3.78 (s, 3H), 4.22 (t, *J* = 7.09 Hz, 2H), 6.58 (s, 1H), 6.85 (t, *J* = 7.58 Hz, 1H) 6.96–7.07 (m, 2H), 7.24 (t, *J* = 7.82 Hz, 1H) 8.30 (s, 1H), (2H under the solvent residual peak); ^13^C NMR (100 MHz, DMSO-*d*_6_) δ = ppm 158.8, 137.3, 129.9, 128.4, 126.8, 121.5, 112.9, 101.4, 56.8, 49.6, 28.6; HRMS (ESI) *m*/*z* calcd. for C_12_H_16_ClN_3_O 218.1215. [M + H]^+^, found 218.1235. 

*1-[2-(3-Methoxyphenyl)ethyl]-1H-imidazol-4-aminehydrochloride* (**7b**): Red oil, quantitative yield; ^1^H NMR (400 MHz, DMSO-*d*_6_) δ = ppm 3.05 (t, *J* = 7.09 Hz, 2H), 3.73 (s, 3H), 4.27 (t, *J* = 7.09 Hz, 2H), 6.64 (s, 1H), 6.73–6.84 (m, 3H), 7.21 (t, *J* = 7.58 Hz, 1H), 8.33 (s, 1 H), (2H under the solvent residual peak); ^13^C NMR (100 MHz, DMSO-*d*_6_) δ = ppm 159.5, 140.7, 137.3, 129.1, 121.4, 115.4, 112.6, 101.4, 56.0, 49.5, 33.8; HRMS (ESI) *m*/*z* calcd. for C_12_H_16_ClN_3_O 218.1215 [M + H]^+^, found 218.1243.

#### 3.2.4. General Procedure for the Synthesis of 6-Chloro-2-methyl-*N*-[1-(2-phenylethyl)-1*H*-imidazol-4-yl]pyrimidin-4-amines **8a**,**b**

Intermediates **7a**,**b** (561 mg, 2.21 mmol) were dissolved in dry DMSO (9 mL). Then 4,6-dichloro-2-methylpyrimidine (396 mg, 2.43 mmol) and DIPEA (1.54 mL, 8.84 mmol) were added and the mixture was heated at 50 °C overnight. Once the reaction was determined to be complete, DIPEA was evaporated, AcOH (1 mL) was added and the resulting solution was loaded on RediSep C18Aq column (150 g) primed with water + 0.1% AcOH. The column was then eluted with 3CV of water + 0.1% AcOH and then the eluent was gradually changed to acetonitrile + 0.1% AcOH over 12CV.

*6-Chloro-N-{1-[2-(2-methoxyphenyl)ethyl]-1H-imidazol-4-yl}-2-methylpyrimidin-4-amine* (**8a**): Purple solid, 55% yield, mp: 135–137 °C; IR: ν = 3273 (NH) cm^−1^; ^1^H NMR (400 MHz, DMSO-*d*_6_) δ = ppm 2.43 (s, 3H), 3.01 (t, *J* = 7.09 Hz, 2H), 3.80 (s, 3H), 4.15 (t, *J* = 7.09 Hz, 2H), 6.69 (br s, 1H), 6.84 (t, *J* = 7.34 Hz, 1H), 6.99 (d, *J* = 8.31 Hz, 1H), 7.07 (dd, *J* = 7.58, 1.71 Hz, 2H), 7.20–7.25 (m, 1H), 7.34 (s, 1H), 10.00 (br s, 1H); ^13^C NMR (100 MHz, DMSO-*d*_6_) δ = ppm 169.7, 158.8, 158.2, 158.1, 154.7, 139.1, 129.9, 128.4, 126.8, 121.5, 112.9, 110.9, 102.7, 56.8, 49.6, 28.6, 25.4; HRMS (ESI) *m*/*z* calcd. for C_17_H_18_ClN_5_O 344.1979 [M + H]^+^, found 344.1991. 

*6-Chloro-N-{1-[2-(3-methoxyphenyl)ethyl]-1H-imidazol-4-yl}-2-methylpyrimidin-4-amine* (**8b**): Purple solid, 57% yield, mp: 137–140 °C; IR: ν = 3273 (NH) cm^−1^; ^1^H NMR (400 MHz, DMSO-*d*_6_) δ = ppm 2.43 (s, 3H), 3.02 (t, *J* = 7.09 Hz, 2H), 3.71 (s, 3H), 4.21 (t, *J* = 7.09 Hz, 2H), 6.73 (br s, 1H), 6.75–6.82 (m, 4H), 7.20 (t, *J* = 8.31 Hz, 1H), 7.38 (s, 1H), 10.00 (br s, 1H); ^13^C NMR (100 MHz, DMSO-*d*_6_) δ = ppm 169.8, 159.5, 158.2, 158.1, 154.6, 140.7, 139.1, 129.1, 121.4, 115.4, 112.6, 110.9, 102.6, 56.0, 49.5, 33.8, 25.4; HRMS (ESI) *m*/*z* calcd. for C_17_H_18_ClN_5_O 344.1979 [M + H]^+^, found 344.1995. 

#### 3.2.5. General Procedure for the Synthesis of 2-Methyl-*N*-[1-(2-phenylethyl)-1*H*-imidazol-4-yl]-6-piperazin-1-ylpyrimidin-4-amines **4a**,**c** and **9a**–**c**

The opportune piperazine (0.73 mmol) and DIPEA (0.10 mL, 0.58 mmol) were added to a solution of the appropriate 6-chloro-2-methyl-*N*-[1-(2-phenylethyl)-1*H*-imidazol-4-yl]pyrimidin-4-amine **8a,b** (100 mg, 0.29 mmol) in DMSO (3 mL). The resulting mixture was heated at 110 °C for 4 h under microwave irradiation. The solution in DMSO was acidified with AcOH (0.5 mL) and then loaded on RediSep C18aq column (50 g) column primed with water + 0.1% AcOH.

To obtain compounds **4a**,**c** and **9c** the column was eluted with 2CV of water + 0.1% AcOH and then the eluent was gradually changed to acetonitrile + 0.1% AcOH over 13CV. The fractions were combined and evaporated. The orange-brown residue was dissolved in DCM and treated with a saturated solution of sodium bicarbonate to avoid the acetic acid salt formation. Finally, the organic phase was dried, filtered and evaporated under vacuum affording the desired compounds. 

To obtain compounds **9a**,**b**, the column was eluted with 3CV of water + 0.1% AcOH and then the eluent was gradually changed to acetonitrile + 0.1% AcOH over 13CV. The fractions containing the desired product were combined and evaporated to obtain a brown residue. It was dissolved in MeOH and loaded onto a SPE-SCX (5 g) eluted first with methanol and then with ammonia in methanol (2N). Basic fractions were collected and evaporated affording the desired compounds.

*2-{4-[6-({1-[2-(3-Methoxyphenyl)ethyl]-1H-imidazol-4-yl}amino)-2-methylpyrimidin-4-yl]piperazin-1-yl}ethanol* (**4a**): Glassy yellow residue, 65% yield; IR: ν = 3600–2825 (NH + OH) cm^−1^; ^1^H NMR (400 MHz, DMSO-*d*_6_) δ = ppm 2.27 (s, 3H), 2.39–2.47 (m, 6H), 3.01 (t, *J* = 7.09 Hz, 2H), 3.41–3.43 (m, 4H), 3.55 (t, *J* = 7.09 Hz, 2H), 3.71 (s, 3H), 4.17 (t, *J* = 7.34 Hz, 2H), 4.40 (t, *J* = 5.38 Hz, 1H), 5.94 (s, 1H), 6.75–6.83 (m, 3H), 7.12–7.23 (m, 2H), 7.28 (s, 1H), 8.89 (s, 1H); ^13^C NMR (100 MHz, DMSO-*d*_6_) δ = ppm 168.9, 159.5, 158.4, 157.3, 154.7, 140.7, 139.1, 129.1, 121.4, 115.4, 112.6, 110.9, 89.5, 59.3, 58.0, 56.0, 52.2, 49.5, 47.5, 33.8, 25.4; HRMS (ESI) *m*/*z* calcd. for C_23_H_31_N_7_O_2_ 438.2539 [M + H]^+^, found 438.2572.

*2-{4-[6-({1-[2-(2-Methoxyphenyl)ethyl]-1H-imidazol-4-yl}amino)-2-methylpyrimidin-4-yl]piperazin-1-yl}ethanol* (**4c**): Glassy yellow residue, 57% yield; IR: ν = 3590–2820 (NH + OH) cm^−1^; ^1^H NMR (400 MHz, DMSO-*d*_6_) δ = ppm 2.27 (s, 3H), 2.40–2.47 (m, 6H), 3.00 (t, *J* = 7.09 Hz, 2H), 3.38–3.44 (m, 4H), 3.53 (t, *J* = 7.09 Hz, 2H), 3.81 (s, 3H), 4.11 (t, *J* = 7.34 Hz, 2H), 4.40 (t, *J* = 5.38 Hz, 1H), 5.93 (s, 1H), 6.81–6.86 (m, 1H), 6.98 (d, *J* = 7.83 Hz, 1H), 7.05–7.12 (m, 2H), 7.19–7.26 (m, 2H), 8.88 (s, 1H); ^13^C NMR (100 MHz, DMSO-*d*_6_) δ = ppm 168.9, 158.8, 158.4, 157.3, 154.7, 139.1, 129.9, 128.4, 126.8, 121.5, 112.9, 110.9, 89.5, 59.3, 58.0, 56.8, 52.2, 49.6, 47.5, 28.6, 25.4; HRMS (ESI) *m*/*z* calcd. for C_23_H_31_N_7_O_2_ 438.2539 [M + H]^+^, found 438.2525.

*N-{1-[2-(2-Methoxyphenyl)ethyl]-1H-imidazol-4-yl}-2-methyl-6-piperazin-1-ylpyrimidin-4-amine* (**9a**): Yellow oil, 72% yield; IR: ν = 3200 (NH) cm^−1^; ^1^H NMR (400 MHz, DMSO-*d*_6_) δ = ppm 2.27 (s, 3H), 2.71–2.73 (m, 4H), 3.00 (t, *J* = 7.09 Hz, 2H), 3.81 (s, 3H), 4.11 (t, *J* = 7.09 Hz, 2H), 5.91 (br s, 1H), 6.85 (d, *J* = 7.34 Hz, 1H), 6.99 (d, *J* = 8.31 Hz, 1H), 7.05–7.13 (m, 2H), 7.19–7.27 (m, 2H), 8.85 (br s, 1H), (5H under the solvent residual peak); ^13^C NMR (100 MHz, DMSO-*d*_6_) δ = ppm 168.9, 158.8, 158.4, 157.3, 154.7, 139.1, 129.9, 128.4, 126.8, 121.5, 112.9, 110.9, 89.5, 56.8, 49.6, 48.1, 44.9, 28.6, 25.4; HRMS (ESI) *m*/*z* calcd. for C_21_H_27_N_7_O 394.2271 [M + H]^+^, found 394.2284. 

*N-{1-[2-(3-Methoxyphenyl)ethyl]-1H-imidazol-4-yl}-2-methyl-6-piperazin-1-ylpyrimidin-4-amine* (**9b**): Yellow oil, 80% yield; IR: ν = 3210 (NH) cm^−1^; ^1^H NMR (400 MHz, DMSO-*d*_6_) δ = ppm 2.27 (s, 3H), 2.71–2.73 (m, 4H), 3.00 (t, *J* = 7.09 Hz, 2H), 3.32–3.35 (m, 4H), 3.71 (s, 3H), 4.17 (t, *J* = 7.09 Hz, 2H), 5.92 (s, 1H), 6.77–6.79 (m, 3H), 7.11–7.23 (m, 2H), 7.28 (s, 1H), 8.86 (br s, 1H), (1H under the solvent residual peak). ^13^C NMR (100 MHz, DMSO-*d*_6_) δ = ppm 168.9, 159.5, 158.4, 157.3, 154.7, 140.7, 139.1, 129.1, 121.4, 115.4, 112.6, 110.9, 89.5, 56.0, 49.5, 48.1, 44.9, 33.8, 25.4. HRMS (ESI) *m*/*z* calcd. for C_21_H_27_N_7_O 394.2271 [M + H]^+^, found 394.2291.

*tert-Butyl4-[6-({1-[2-(3-methoxyphenyl)ethyl]-1H-imidazol-4-yl}amino)-2-methylpyrimidin-4-yl]piperazine-1-carboxylate* (**9c**): Pink solid, 28% yield, mp: 145–147 °C; IR: ν = 3600 (NH) cm^−1^; ^1^H NMR (400 MHz, DMSO-*d*_6_) δ = ppm 1.43 (s, 9H), 2.29 (s, 3H), 2.94 (t, *J* = 7.09 Hz, 2H), 3.36–3.46 (m, 8H), 3.73 (s, 3H), 4.14 (t, *J* = 7.09 Hz, 2H), 5.95 (s, 1H), 6.60–6.65 (m, 3H), 7.05–7.09 (m, 1H), 7.14 (s, 1H), 7.29 (d, *J* = 1.47 Hz, 1H), 9.27 (s, 1H); ^13^C NMR (100 MHz, DMSO-*d*_6_) δ = ppm 168.99, 159.4, 158.3, 157.3, 155.1, 154.6, 140.7, 139.1, 129.1, 121.4, 115.4, 112.6, 110.9, 89.5, 80.9, 56.0, 49.5, 47.8, 43.6, 33.8, 28.4, 25.4; HRMS (ESI) *m*/*z* calcd. for C_26_H_35_N_7_O_3_ 494.2801 [M + H]^+^, found 494.2837.

#### 3.2.6. General Procedure for the Synthesis of Methyl [4-(2-methyl-6-{[1-(2-phenylethyl)-1*H*-imidazol-4-yl]amino}pyrimidin-4-yl)piperazin-1-yl]acetate Derivatives **10a**,**b**

A mixture of the opportune 2-methyl-*N*-[1-(2-phenylethyl)-1*H*-imidazol-4-yl]-6-piperazin-1-ylpyrimidin-4-amine **9a,b** (79 mg, 0.20 mmol), methyl bromoacetate (19 μL, 0.20 mmol), TEA (28 μL, 0.20 mmol) in DMF (1.5 mL) was stirred at room temperature for 10min [27]. DMF was evaporated under reduced pressure and the crude was loaded onto a Biotage KP-NH (28 g) column primed with CyHex/AcOEt 1:1. The column was then run for 2CV with CyHex/EtOAc 1:1 and then changed to AcOEt only for 4CV. 

*Methyl{4-[6-({1-[2-(2-methoxyphenyl)ethyl]-1H-imidazol-4-yl}amino)-2-methylpyrimidin-4-yl]piperazin-1-yl}acetate* (**10a**): Pale yellow residue, 72% yield; IR: ν = 3200 (NH), 1745 (CO) cm^−1^; ^1^H NMR (400 MHz, DMSO-*d*_6_) δ = ppm 2.28 (s, 3H), 2.52–2.57 (m, 6H), 3.00 (t, *J* = 7.09 Hz, 2H), 3.42–3.46 (m, 4H), 3.63 (s, 3H), 3.81 (s, 3H), 4.11 (t, *J* = 7.09 Hz, 2H), 5.94 (s, 1H), 6.84 (t, *J* = 7.34 Hz, 1H), 6.99 (d, *J* = 8.31 Hz, 1H), 7.06–7.13 (m, 2H), 7.20–7.27 (m, 2H), 8.90 (br s, 1H); ^13^C NMR (100 MHz, DMSO-*d*_6_) δ = ppm 171.4, 168.9, 158.7, 158.5, 157.3, 154.7, 139.1, 129.9, 128.4, 126.8, 121.5, 112.9, 110.9, 89.5, 60.7, 56.7, 51.8, 51.5, 49.6, 47.5, 28.6, 25.5; HRMS (ESI) *m*/*z* calcd. for C_24_H_31_N_7_O_3_ 466.2486 [M + H]^+^, found 466.2499.

*Methyl{4-[6-({1-[2-(3-methoxyphenyl)ethyl]-1H-imidazol-4-yl}amino)-2-methylpyrimidin-4-yl]piperazin-1-yl}acetate* (**10b**): Pale yellow residue, 56% yield; IR: ν = 3200 (NH), 1735 (CO) cm^−1^; ^1^H NMR (400 MHz, DMSO-*d*_6_) δ = ppm 2.28 (s, 3H), 2.53–2.57 (m, 6H), 3.01 (t, *J* = 7.09 Hz, 2H), 3.41–3.45 (m, 4H), 3.63 (s, 3H), 3.71 (s, 3H), 4.17 (t, *J* = 7.09 Hz, 2H), 5.94 (br s, 1H), 6.75–6.84 (m, 3H), 7.12–7.23 (m, 2H), 7.28 (s, 1H), 8.90 (br s, 1H); ^13^C NMR (100 MHz, DMSO-*d*_6_) δ = ppm 171.4, 168.9, 159.5, 158.4, 157.3, 154.7, 140.7, 139.1, 129.1, 121.4, 115.4, 112.6, 110.9, 89.5, 60.7, 56.0, 51.8, 51.5, 49.5, 47.5, 33.8, 25.3; HRMS (ESI) *m*/*z* calcd. for C_24_H_31_N_7_O_3_ 466.2486 [M + H]^+^, found 466.2502. 

#### 3.2.7. General Procedure for the Synthesis of 2-[4-(2-Methyl-6-{[1-(2-phenylethyl)-1*H*-imidazol-4-yl]amino}pyrimidin-4-yl)piperazin-1-yl]acetamide Derivatives **4b**,**d**

Ammonia 7N in MeOH (10 mL, 70.0 mmol) was added to the opportune methyl [4-(2-methyl-6-{[1-(2-phenylethyl)-1*H*-imidazol-4-yl]amino}pyrimidin-4-yl)piperazin-1-yl]acetate derivative **10a**,**b** (102 mg, 0.22 mmol). The mixture was sealed and heated at 100 °C under microwave irradiation for 3 h [27]. The reaction was cooled to room temperature and then solvent was evaporated under reduced pressure. The crude was dissolved in DMSO and AcOH. The resulting solution was on then loaded on RediSep C18aq column (50 g) column primed with water + 0.1% AcOH. The column was then eluted with 3CV of water + 0.1% AcOH and then the eluent was gradually changed to acetonitrile + 0.1% AcOH over 13CV. The fractions containing the desired product were then combined and evaporated to obtain a yellow residue. It was dissolved in MeOH and loaded onto a SPE-SCX (2 g), eluted first with methanol and then with ammonia in MeOH (2N). Basic fractions were collected and evaporated to obtain desired compounds **4b**,**d**.

*2-{4-[6-({1-[2-(3-Methoxyphenyl)ethyl]-1H-imidazol-4-yl}amino)-2-methylpyrimidin-4-yl]piperazin-1-yl}acetamide* (**4b**): white solid, 57% yield, mp: 198–200 °C; IR: ν = 3260, 3180 (NH_2_), 1675 (CO) cm^−1^; ^1^H NMR (400 MHz, DMSO-*d*_6_) δ = ppm 2.28 (s, 3H), 2.91 (br s, 2H), 3.01 (t, *J* = 7.09 Hz, 2H), 3.44–3.48 (m, 4H), 3.71 (s, 3H), 4.17 (t, *J* = 7.09 Hz, 2H), 5.95 (s, 1H), 6.76–6.81 (m, 3H), 7.09–7.26 (m, 4H), 7.29 (d, *J* = 1.47 Hz, 1H), 8.91 (br s, 1H), (4H under the solvent residual peak); ^13^C NMR (100 MHz, DMSO-*d*_6_) δ = ppm 169.2, 168.9, 159.4, 158.4, 157.3, 154.7, 140.7, 139.3, 129.1, 121.4, 115.4, 112.5, 110.9, 89.5, 60.6, 56.0, 51.5, 49.5, 47.5, 33.8, 25.4; HRMS (ESI) *m*/*z* calcd. for C_23_H_30_N_8_O_2_ 451.2488 [M + H]^+^, found 451.2510.

*2-{4-[6-({1-[2-(2-Methoxyphenyl)ethyl]-1H-imidazol-4-yl}amino)-2-methylpyrimidin-4-yl]piperazin-1-yl}acetamide* (**4d**): White solid, 69% yield, mp: 195–198 °C; IR: ν = 3260, 3180 (NH_2_), 1675 (CO) cm^−1^; ^1^H NMR (400 MHz, DMSO-*d*_6_) δ = ppm 2.28 (s, 3H), 2.91 (br s, 2H), 3.01 (t, *J* = 7.09 Hz, 2H), 3.44–3.48 (m, 4H), 3.71 (s, 3H), 4.17 (t, *J* = 7.09 Hz, 2H), 5.95 (s, 1H), 6.76–6.81 (m, 2H), 7.09–7.26 (m, 6H), 7.28–7.30 (m, 1H), 8.91 (br s, 1H), (3H under the solvent residual peak); ^13^C NMR (100 MHz, DMSO-*d*_6_) δ = ppm 169.2, 168.9, 158.7, 158.4, 157.3, 154.7, 139.1, 129.9, 128.4, 126.8, 121.5, 112.9, 110.9, 89.5, 60.5, 56.8, 51.5, 49.6, 47.4, 28.6, 25.4; HRMS (ESI) *m*/*z* calcd. for C_23_H_30_N_8_O_2_ 451.2488 [M + H]^+^, found 451.2502.

#### 3.2.8. General Procedure for the Synthesis of (2-{4-[(2-Methyl-6-piperazin-1-ylpyrimidin-4-yl)amino]-1*H*-imidazol-1-yl}ethyl)phenol Derivatives **4e**–**l**

Sodium methanethiolate (91 mg, 1.30 mmol to obtain compounds **4e**–**g**, **4j**–**l**; 98 mg, 1.40 mmol to obtain compounds **4h**,**i**) was added to a solution of the appropriate *N*-{1-[2-(methoxyphenyl)ethyl]-1*H*-imidazol-4-yl}-2-methyl-6-piperazin-1-ylpyrimidin-4-amine derivative **4a**–**d**, **9a**–**c**, **10b** (0.10 mmol) in DMF (2 mL). The mixture was bubbled with N_2_ for 3min and heated at 140 °C for 2–6 h under microwave irradiation. The reaction was cooled to room temperature, AcOH/water (1:1) was added until pH 5/6 and then the mixture was evaporated to dryness under Rxn RP (2 g) cartridges high vacuum. The residue obtained was redissolved in DMF and loaded onto PoraPakTM primed with water + 0.1% AcOH. The column was run with water + 0.1% AcOH for 3CV and then the eluent was gradually changed to acetonitrile + 0.1% AcOH for 10CV. Fractions containing the desired product were evaporated to give compounds **4g**, **j**–**l**. To obtain compounds **4e**,**f**,**h**,**i**, the residue was dissolved in MeOH and loaded onto a SPE-SCX (2 g) eluted first with MeOH and then with ammonia in MeOH (2N). Basic fractions were collected and evaporated giving the desired compounds.

*2-{2-[4-({6-[4-(2-Hydroxyethyl)piperazin-1-yl]-2-methylpyrimidin-4-yl}amino)-1H-imidazol-1-yl]ethyl}phenol* (**4e**): Pale yellow solid, 30% yield, mp: 151–153 °C; IR: ν = 3500–3250 (2OH, NH) cm^−1^; ^1^H NMR (400 MHz, DMSO-*d*_6_) δ = ppm 2.28 (s, 3H), 2.97 (t, *J* = 7.09 Hz, 2H), 3.35–3.49 (m, 3H), 3.58 (m, 2H), 4.08 (d, *J* = 5.38 Hz, 1H), 4.12 (t, *J* = 7.09 Hz, 2H), 4.45 (br s, 1H), 5.97 (br s, 1H), 6.69 (t, *J* = 7.46 Hz, 1H), 6.80–6.84 (m, 1H), 6.98–7.07 (m, 2H), 7.13 (br s, 1H), 7.29 (d, *J* = 1.47 Hz, 1H), 8.95 (br s, 1H), 9.46 (s, 1H), (6H under the solvent residual peak); ^13^C NMR (100 MHz, DMSO-*d*_6_) δ = ppm 168.9, 158.4, 157.3, 154.8, 154.7, 139.1, 129.3, 126.7, 124.3, 120.9, 116.7, 110.9, 89.5, 59.3, 58.0, 52.2, 49.6, 47.5, 28.8, 25.4; HRMS (ESI) *m*/*z* calcd. for C_22_H_29_N_7_O_2_ 424.2372 [M + H]^+^, found 424.2401.

*2-(2-{4-[(2-Methyl-6-piperazin-1-ylpyrimidin-4-yl)amino]-1H-imidazol-1-yl}ethyl)phenol* (**4f**): White solid, 24% yield, mp: 193–196 °C; IR: ν = 3595–3300 (OH, NH) cm^−1^; ^1^H NMR (400 MHz, DMSO-*d*_6_) δ = ppm 2.27 (s, 3H), 2.74–2.79 (m, 4H), 2.94 (t, *J* = 7.09 Hz, 2H), 3.35–3.40 (m, 4H), 4.14 (t, *J* = 7.09 Hz, 2H), 5.91–5.95 (m, 1H), 6.66–6.71 (m, 1H), 6.80–6.85 (m, 1H), 6.99–7.07 (m, 2H), 7.12 (s, 1H), 7.28 (s, 1H), 8.86–8.90 (m, 2H), 9.46 (s, 1H); ^13^C NMR (100 MHz, DMSO-*d*_6_) δ = ppm 168.9, 158.4, 157.3, 154.8, 154.7, 139.1, 129.3, 126.7, 124.3, 120.9, 116.7, 110.9, 89.5, 49.6, 48.1, 44.9, 28.8, 25.4; HRMS (ESI) *m*/*z* calcd. for C_20_H_25_N_7_O 380.2020 [M + H]^+^, found 380.2045.

*2-{4-[6-({1-[2-(2-Hydroxyphenyl)ethyl]-1H-imidazol-4-yl}amino)-2-methylpyrimidin-4-yl]piperazin-1-yl}acetamide* (**4g**): pale yellow solid, 30% yield, mp: 160–163 °C; IR: ν = 3590 (OH), 3520 (NH), 3363 (NH), 1640 (CO) cm^−1^; ^1^H NMR (400 MHz, DMSO-*d*_6_) δ = ppm 2.28 (s, 3H), 2.43–2.48 (m, 2H), 2.90 (s, 2H), 2.97 (t, *J* = 7.34 Hz, 2H), 3.47 (m, 4H), 4.12 (t, *J* = 7.34 Hz, 2H), 5.95 (s, 1H), 6.69 (s, 1H), 6.82 (d, *J* = 7.34 Hz, 1H), 6.98–7.07 (m, 2H), 7.12 (br s, 2H), 7.23 (br s, 1H), 7.28 (d, *J* = 1.47 Hz, 1H), 8.90 (br s, 1H), 9.48 (br s, 1H), (2H under the solvent residual peak); ^13^C NMR (100 MHz, DMSO-*d*_6_) δ = ppm 169.2, 168.9, 158.4, 157.3, 154.8, 154.7, 139.1, 129.3, 126.7, 124.3, 120.9, 116.7, 110.9, 89.5, 60.6, 51.5, 49.6, 47.4, 28.8, 25.3; HRMS (ESI) *m*/*z* calcd. for C_22_H_28_N_8_O_2_ 437.2330 [M + H]^+^, found 437.2373.

*3-{2-[4-({6-[4-(2-Hydroxyethyl)piperazin-1-yl]-2-methylpyrimidin-4-yl}amino)-1H-imidazol-1-yl]ethyl}phenol* (**4h**): White solid, 20% yield, mp: 150–155 °C; IR: ν = 3500–3250 (2OH, NH) cm^−1^; ^1^H NMR (400 MHz, DMSO-*d*_6_) δ = ppm 2.28 (s, 3H), 2.44–2.48 (m, 4H), 2.94 (t, *J* = 7.34 Hz, 2H), 3.40–3.43 (m, 4H), 3.50–3.58 (m, 2H), 4.13 (t, *J* = 7.34 Hz, 2H), 4.41 (br s, 1H), 5.94 (s, 1H), 6.59–6.66 (m, 3H), 7.04–7.10 (m, 1H), 7.14 (s, 1H), 7.29 (d, *J* = 1.47 Hz, 1H), 8.89 (br s, 1H), 9.26 (s, 1H), (2H under the solvent residual peak); ^13^C NMR (100 MHz, DMSO-*d*_6_) δ = ppm 168.9, 158.4, 157.8, 157.3, 154.7, 140.8, 139.1, 130.3, 120.7, 116.7, 116.2, 110.9, 89.5, 59.3, 58.0, 52.2, 49.5, 47.4, 33.8, 25.5; HRMS (ESI) *m*/*z* calcd. for C_22_H_29_N_7_O_2_ 424.2372 [M + H]^+^, found 424.2411.

*3-(2-{4-[(2-Methyl-6-piperazin-1-ylpyrimidin-4-yl)amino]-1H-imidazol-1-yl}ethyl)phenol* (**4i**): Yellow solid, 35% yield, mp: 193–196 °C; IR: ν = 3590 (OH), 3363 (NH), 3300 (NH) cm^−1^; ^1^H NMR (400 MHz, DMSO-*d*_6_) δ = ppm 2.27 (s, 3H), 2.72–2.76 (m, 4H), 2.94 (t, *J* = 7.09 Hz, 2H), 3.34–3.38 (m, 4H), 4.13 (t, *J* = 7.09 Hz, 2H), 5.92 (s, 1H), 6.59–6.65 (m, 3H), 7.04–7.16 (m, 3H), 7.29 (d, *J* = 1.47 Hz, 1H), 8.86 (br s, 1H), 9.26 (s, 1H); ^13^C NMR (100 MHz, DMSO-*d*_6_) δ = ppm 168.9, 158.4, 157.8, 157.3, 154.7, 140.8, 139.1, 130.3, 120.7, 116.7, 116.2, 110.9, 89.5, 49.5, 48.1, 44.9, 33.8, 25.3; HRMS (ESI) *m*/*z* calcd. for C_20_H_25_N_7_O 380.2021 [M + H]^+^, found 380.2060.

*2-{4-[6-({1-[2-(3-Hydroxyphenyl)ethyl]-1H-imidazol-4-yl}amino)-2-methylpyrimidin-4-yl]piperazin-1-yl}acetamide* (**4j**): Glassy yellow residue, 28% yield; IR: ν = 3580 (OH), 3525 (NH), 3363 (NH), 1645 (CO) cm^−1^; ^1^H NMR (400 MHz, DMSO-*d*_6_) δ = ppm 2.28 (s, 3H), 2.46–2.49 (m, 2H), 2.90 (s, 2H), 2.94 (t, *J* = 7.09 Hz, 2H), 3.44–3.49 (m, 4H), 4.14 (t, *J* = 7.09 Hz, 2H), 5.92–5.96 (m, 1H), 6.59–6.65 (m, 3H), 7.04–7.09 (m, 1H), 7.14 (br s, 2H), 7.22 (br s, 1H), 7.29 (d, *J* = 1.47 Hz, 1H), 8.89 (br s, 1H), 9.27 (br s, 1H), (2H under the solvent residual peak); ^13^C NMR (100 MHz, DMSO-*d*_6_) δ = ppm 169.2, 168.9, 158.4, 157.8, 157.3, 154.7, 140.8, 139.1, 130.3, 120.7, 116.7, 116.2, 110.9, 89.5, 60.5, 51.5, 49.5, 47.4, 33.8, 25.4; HRMS (ESI) *m*/*z* calcd. for C_22_H_28_N_8_O_2_ 437.2332 [M + H]^+^, found 437.2379.

*Methyl{4-[6-({1-[2-(3-hydroxyphenyl)ethyl]-1H-imidazol-4-yl}amino)-2-methylpyrimidin-4-yl]piperazin-1-yl}acetate* (**4k**): White solid, 67% yield, mp: 127–130 °C; IR: ν = 3590 (OH), 3360 (NH); 1745 (CO) cm^−1^; ^1^H NMR (400 MHz, DMSO-*d*_6_) δ = ppm 2.28 (s, 3H), 2.55–2.57 (m, 4H), 2.94 (t, *J* = 7.34 Hz, 2H), 3.29 (s, 2H), 3.41–3.46 (m, 4H), 3.63 (s, 3H), 4.13 (t, *J* = 7.34 Hz, 2H), 5.94 (s, 1H), 6.59–6.65 (m, 3H), 7.04–7.09 (m, 1H), 7.14 (s, 1H), 7.29 (d, *J* = 1.47 Hz, 1H), 8.89 (s, 1H), 9.28 (br s, 1H); ^13^C NMR (100 MHz, DMSO-*d*_6_) δ = ppm 171.4, 168.9, 158.4, 157.8, 157.3, 154.7, 140.8, 139.1, 130.3, 120.7, 116.7, 116.2, 110.9, 89.5, 60.7, 51.8, 51.5, 49.5, 47.4, 33.8, 25.5; HRMS (ESI) *m*/*z* calcd. for C_23_H_29_N_7_O_3_ 452.2332 [M + H]^+^, found 452.2359. 

*tert-Butyl4-[6-({1-[2-(3-hydroxyphenyl)ethyl]-1H-imidazol-4-yl}amino)-2-methylpyrimidin-4-yl]piperazine-1-carboxylate* (**4l**): pale pink solid, 44% yield, mp: 127–130 °C; IR: ν = 3595 (OH), 3363 (NH), 1750 (CO) cm^−1^; ^1^H NMR (400 MHz, DMSO-*d*_6_) δ = ppm 1.43 (s, 9H), 2.29 (s, 3H), 2.94 (t, *J* = 7.09 Hz, 2H), 3.36–3.46 (m, 8H), 4.14 (t, *J* = 7.09 Hz, 2H), 5.95 (s, 1H), 6.60–6.65 (m, 3H), 7.05–7.09 (m, 1H), 7.14 (s, 1H), 7.29 (d, *J* = 1.47 Hz, 1H), 8.96 (s, 1H), 9.27 (s, 1H); ^13^C NMR (100 MHz, DMSO-*d*_6_) δ = ppm 168.9, 158.4, 157.8, 157.3, 155.1, 154.7, 140.8, 139.1, 130.3, 120.7, 116.7, 116.2, 110.9, 89.5, 80.9, 49.5, 47.8, 47.8, 43.6, 33.8, 28.5, 28.4, 25.4; HRMS (ESI) *m*/*z* calcd. for C_25_H_33_N_7_O_3_ 480.2651 [M + H]^+^, found 480.2610.

### 3.3. Enzymatic Assays

Enzymatic assays to determine the kinase inhibitory activity of NCEs were performed using an automatic liquid-handling device (Microlab STAR Hamilton) and Z’-LYTE Kinase Assay Platform, a fluorescence resonance energy transfer (FRET)-based assay platform compatible with high-throughput screening (HTS) applications. The assay has a fluorescence-based, coupled-enzyme format and uses the differential sensitivities of phosphorylated and non-phosphorylated peptides toward proteolytic cleavage. The assay procedure was carried out according to the supplier’s indications. Test compounds were evaluated in a primary screen against active human recombinant Src (62.3 kDa), followed by a profiling assay toward a selected panel of Src family kinases (Fyn, LynA, and Yes1, all human recombinant full-length proteins) according to a procedure already reported. Final results have been expressed as percent inhibition, and IC_50_ values were calculated by non-linear curve fitting using GraphPad Prism software (version 6 for Windows). The inter-experimental variability of IC_50_ values resulted within accepted limits of ±0.5 log units.

### 3.4. Cellular Assays 

In vitro experiments were carried out using human neuroblastoma cell line SH-SY5Y, human glioblastoma cell line U-87, and human erythroleukemia cell line K-562. Cell lines were obtained from American Tissue Culture Collection (ATCC, SH-SY5Y CRL-2266; U-87 HTB-14; K-562 CCL-243). K-562 cells were cultured in RPMI medium with 10% FCS. SH-SY5Y and U-87 cells were cultured in DMEM medium with 10% FCS. In order to determine antiproliferative effect of NCE compounds cells were seeded at density of 5 × 10^4^ cells/mL (K-562) or 10 × 10^4^ cells/cm^2^ (SH-SY5Y, U-87) and treated with increasing concentrations of NCE compounds. Control cells were treated with the vehicle of the experimental point containing the highest percentage of DMSO. Cell cultures were maintained at 37 °C in 5% *v*/*v* CO_2_ for 72 h. Cell number and vitality were evaluated on cell suspension using the automatic cell counter NucleoCounter^®^ (Chemometec, Denmark). Results from the NucleoCounter represented either total or non-viable cell concentration, depending on the sample preparation indicated by the manufacturer. Each experiment was performed at least three times and results were expressed as mean and standard deviation. 

## Figures and Tables

**Table 1:**
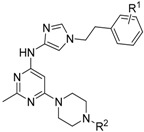
NCEs activities and docking scores.

Cmpd	R^1^	R^2^	Src Docking Score (kcal/mol)	Src IC_50_ (nM) ^a^	Fyn IC_50_ (nM) ^a^	Lyn IC_50_ (nM) ^a^	Yes IC_50_ (nM) ^a^
**dasat.**				<4 ^b^	<9 ^b^	<0.9 ^b^	<3 ^b^
**2**	H	-CH_2_CH_2_OH	−10.02	220 ±0.03	689 ± 0.10	1300 ± 0.02	167 ± 0.03
**4a**	-3OMe	-CH_2_CH_2_OH	−10.10	260 ± 23	146 ± 30	451 ± 13	560 ± 40
**4b**	-3OMe	-CH_2_CONH_2_	−7.26	1476 ± 185	1454 ± 768	1808 ± 307	1843 ± 812
**4c**	-2OMe	-CH_2_CH_2_OH	−10.58	225 ± 8	439 ± 45	145 ± 31	55 ± 9
**4d**	-2OMe	-CH_2_CONH_2_	−8.33	1533 ± 44	1387 ± 93	1205 ± 189	NA
**4e**	-2OH	-CH_2_CH_2_OH	−10.97	59 ± 5	73 ± 5	17 ± 0.54	11 ± 0.6
**4f**	-2OH	-H	−10.98	56 ± 5	50 ± 4	51 ± 3	3 ± 1
**4g**	-2OH	-CH_2_CONH_2_	−11.23	40 ± 5	40 ± 5	19 ± 2	15 ± 4
**4h**	-3OH	-CH_2_CH_2_OH	−10.80	93 ± 19	10 ± 0.4	20 ± 0.8	3 ± 1
**4i**	-3OH	-H	−10.68	83 ± 18	10 ± 0.4	27 ± 1	3 ± 0.8
**4j**	-3OH	-CH_2_CONH_2_	−11.33	40 ± 3	12 ± 2	10 ± 1.2	3 ± 0.7
**4k**	-3OH	-CH_2_COOMe	−10.99	40 ± 2	9 ± 1	18 ± 1	3 ± 1
**4l**	-3OH	-COO*tert*-butyl	−10.99	50 ± 3	14 ± 1.5	26 ± 3	7 ± 0.35

^a^ The compound was tested in two independent experiments, and IC_50_ values are the mean ± SD. ^b^ Dasatinb was used as reference; the IC_50_ values of dasatinib were less than that of enzyme concentrations, which were 4, 9, 0.9, and 3 nm for Src, Fyn, Lyn, and Yes, respectively.

## Supplementary Materials

The following are available online. Figure S1: Antiproliferative effect of selected compounds evaluated by in vitro analysis of percentage of viable cells respect untreated cells (100%) after 72 h of incubation. Compounds were used for treating neuroblastoma cells (upper panel) and glioblastoma cells (bottom panel).

## Abbreviations

ABCB1	ATP-binding cassette sub-family B member 1
ALL	acute lymphoblastic leukemia
Bcr-Abl	breakpoint cluster region-Abelson
Btk	Bruton’s tyrosine kinase
CML	chronic myeloid leukemia
c-Src	cellular-sarcoma tyrosine kinase
DIPEA	*N*,*N*-diisopropylethylamine
EMA	European Medicines Agency
FDA	Food and Drug Admnistration
FRET	fluorescence resonance energy transfer
GBM	glioblastoma multiforme
HRI	hydrophobic region I
HTS	high-throughput screening
NB	neuroblastoma
NCEs	new chemical entities
PDGFR	platelet derived growth factor receptor
Ph^+^	Philadelphia chromosome–positive
RET	REarranged during Transfection
SFK	Src family kinase
SH	Src homology
STK	serine/threonine kinase
TK	tyrosine kinase
VEGFR	vascular endothelial growth factor
