# Supplementary material for: Optimization of Aminoimidazole Derivatives as Src Family Kinase Inhibitors
Source: Molecules. 2018 Sep 17;23(9):2369. doi: 10.3390/molecules23092369 (PMC6225123; doi:10.3390/molecules23092369)
Supplement: Supplementary file 1 [file molecules-23-02369-s001.pdf]

Article

Optimization of Aminoimidazole Derivatives as Src Family Kinase Inhibitors

Cinzia Maria Francini[1,2], Francesca Musumeci[3], Anna Lucia Fallacara[4], Lorenzo Botta[5], Alessio Molinari[4], Roberto Artusi[1], Laura Mennuni[1], Adriano Angelucci[6] and Silvia Schenone[3*]

[1] Rottapharm Biotech S.r.l. Valosa di Sopra N9Street, Monza 20900, Italy; cinziamariafrancini@libero.it (C.M.F.); roberto.artusi@rottapharmbiotech.com (R.A.), laura.mennuni@rottapharmbiotech.com (L.M.)
[2] Department of Pharmacy, University of Genova, Viale Benedetto XV 3, Genova 16132, Italy; francesca.musumeci@unige.it (F.M.), schenone@difar.unige.it (S.S.)
[3] Department of Biotechnology, Chemistry and Pharmacy, Via Aldo Moro 2, Siena 53100, Italy; al.fallacara@gmail.com (A.L.F.), molinari.a2@gmail.com (A.M.)
[4] Department of Biological and Ecological Science, University of Tuscia, 01100 Viterbo, Italy; lorenzo.botta@unitus.it
[5] Department of Applied Clinical Science and Biotechnology, University of L'Aquila, Via Vetoio Coppito, L'Aquila 67100, Italy; adriano.angelucci@univaq.it
[†] Present address: Drug Design & Discovery, Aptuit (Verona) Srl, an Evotec Company, Via Alessandro Fleming 4, Verona 37135, Italy
* Correspondence: schenone@difar.unige.it, Tel.: +39 010 3538362

Received: 12 July 2018; Accepted: 13 September 2018; Published: date

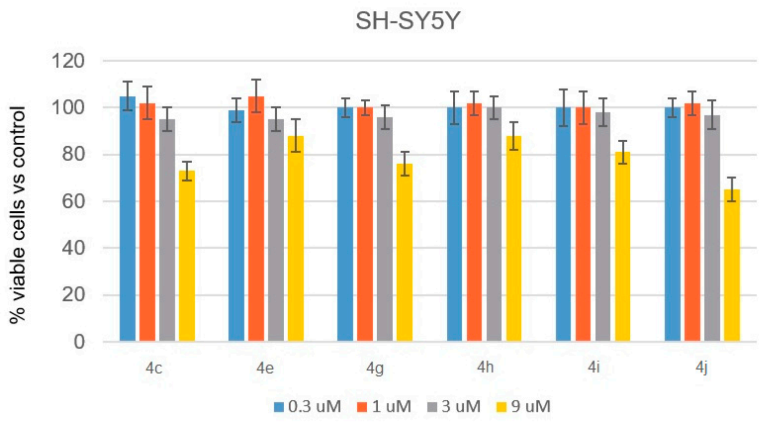

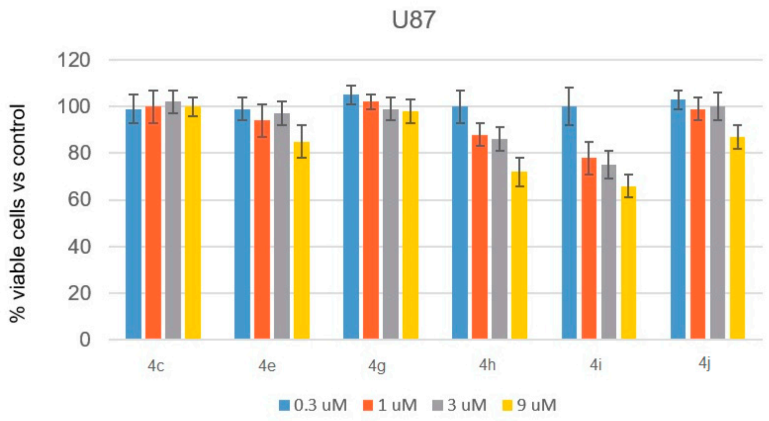

Figure S1. Antiproliferative effect of selected compounds evaluated by in vitro analysis of percentage of viable cells respect untreated cells (100%) after 72 h of incubation. Compounds were used for treating neuroblastoma cells (upper panel) and glioblastoma cells (bottom panel).
